# MINC 2.0: A Flexible Format for Multi-Modal Images

**DOI:** 10.3389/fninf.2016.00035

**Published:** 2016-08-11

**Authors:** Robert D. Vincent, Peter Neelin, Najmeh Khalili-Mahani, Andrew L. Janke, Vladimir S. Fonov, Steven M. Robbins, Leila Baghdadi, Jason Lerch, John G. Sled, Reza Adalat, David MacDonald, Alex P. Zijdenbos, D. Louis Collins, Alan C. Evans

**Affiliations:** ^1^McConnell Brain Imaging Centre, Montreal Neurological Institute, McGill UniversityMontreal, QC, Canada; ^2^Intelerad Medical SystemsMontreal, QC, Canada; ^3^Center for Advanced Imaging, The University of QueenslandBrisbane, QLD, Australia; ^4^Mouse Imaging Centre, The Hospital for Sick ChildrenToronto, ON, Canada; ^5^Department of Medical Biophysics, University of TorontoToronto, ON, Canada; ^6^Autodesk Inc.Montreal, QC, Canada; ^7^Biospective Inc.Montreal, QC, Canada; ^8^Department of Biomedical Engineering, McGill UniversityMontreal, QC, Canada

**Keywords:** neuroimaging, provenance, metadata, data management, data format, HDF5

## Abstract

It is often useful that an imaging data format can afford rich metadata, be flexible, scale to very large file sizes, support multi-modal data, and have strong inbuilt mechanisms for data provenance. Beginning in 1992, MINC was developed as a system for flexible, self-documenting representation of neuroscientific imaging data with arbitrary orientation and dimensionality. The MINC system incorporates three broad components: a file format specification, a programming library, and a growing set of tools. In the early 2000's the MINC developers created MINC 2.0, which added support for 64-bit file sizes, internal compression, and a number of other modern features. Because of its extensible design, it has been easy to incorporate details of provenance in the header metadata, including an explicit processing history, unique identifiers, and vendor-specific scanner settings. This makes MINC ideal for use in large scale imaging studies and databases. It also makes it easy to adapt to new scanning sequences and modalities.

## 1. Introduction

Emerging in 1995 as a formal discipline, human brain mapping has become an indispensable research methodology in numerous clinical and basic research projects that study various populations using neuroimaging. The neuroimaging data collected in a typical cohort study is multi-spectral and multi-resolution, thus contains a rich set of meta-data related to scanning parameters or study design. These data will ultimately be integrated with myriad subject-specific behavioral, biometric, and genetic variables. Because many such studies are longitudinal, and the data is provided through multi-source channels, both pipelined analysis and provenance tracking are essential.

The MINC format for neuroinformatics data was designed and implemented to support this vision, beginning in 1992. The goal of the project was the development of a data format and programming tools for neuroimaging that included several features that remain fairly novel today:

- An extensible header format that includes both data acquisition and analysis history.
- Self-documenting metadata using human-readable, descriptive variable and attribute names.
- Support for high dimensionality and arbitrary coordinate systems, including irregularly sampled dimensions.
- Straightforward support for new modalities.
- Data portability and platform independence.

This vision led to development of the first normative MRI Atlas of human brain (Collins et al., 1994) that has set the standard for developing more modern atlases such as the BigBrain (Amunts et al., 2013).

The original design of MINC (“MINC 1.0”) relied on the NetCDF (Network Common Data Format) library, developed by NCAR primarily for atmospheric data (Rew and Davis, 1990). This gave rise to the acronym MINC for “Medical Imaging NetCDF.” While NetCDF was a huge step forward in terms of the supported data structures, it was not a fully hierarchical system in that it did not support arbitrary nesting of data and metadata.

More recent versions of the MINC format (“MINC 2.0”) have been re-developed to use HDF5 (The HDF Group, 1997–2015). In so doing, it has taken advantage of HDF5's support for hierarchical structure, internal compression, 64-bit file sizes, and other more modern features (Vincent et al., 2004).

Today, the MINC data format and tools are used in several analytical pipelines for functional and anatomical studies such as PSOM (Bellec et al., 2012), CIVET (Ad-Dab'bagh et al., 2006), and FreeSurfer (Fischl, 2012) for cortical thickness analysis. It is implemented in several data processing platforms such as LONI (Rex et al., 2003) and CBRAIN (Sherif et al., 2014) and is available to large-scale data-sharing and management projects served by LORIS (Das et al., 2012) and ADNI (Mueller et al., 2005). MINC is released as open source, under a non-restrictive license agreement and developments to incorporate it into recent Neuroimaging Data Model (NI-DM) initiatives is ongoing.

This paper gives an overview of the history and also the current design and motivation for the MINC 2.0 file format. We will concentrate on high-level design principles to demonstrate how they make MINC extensible to address issues related to data fusion and multivariate modeling.

## 2. Historical motivation and existing solutions

Prior to the creation of MINC, the majority of neuroimaging studies used file formats developed for specific imaging equipment. The community rapidly converged on the format developed for the tool *Analyze 7.5* (Robb et al., 1989), which was co-opted by a number of other tools for functional and structural imaging analysis. Analyze format originally used a pair of files, a fixed-size binary header file (.HDR) that contained a limited set of numeric fields describing a separate binary image file (.IMG). The format was conceptually very simple and could be readily implemented in any programming language.

Almost immediately, the neuroimaging community began to create extensions to the Analyze format to accommodate new and different analysis parameters and imaging metadata. The result was a number of often incompatible variations on the Analyze format. The problem was exacerbated by the fixed binary structure of the Analyze header file, which precluded a standard extension mechanism or flexible labeling of novel data fields.

The DICOM format (Bidgood and Horii, 1992; National Electrical Manufacturers Association, 1993–2015), the successor to the ACR-NEMA format (ACR-NEMA committee, 1985, 1988), was the dominant alternative format, used by medical equipment manufacturers to facilitate the transfer of data from scanners. As designed, DICOM was very powerful, but in practical terms the standardization of the format suffered as vendors relied on proprietary extensions to support 3D images and newer modalities such as functional imaging. Also, because DICOM data fields are identified by short numeric codes, the format is not self-documenting, but instead relies on a lengthy set of manuals to provide descriptions of every field. Vendors make extensive use of both private DICOM fields and introduce complex, undocumented data structures embedded within DICOM (Siemens AG, 2012).

MINC was designed to meet the needs of researchers, while avoiding the limitations and pitfalls of formats such as Analyze and DICOM. Each MINC file represents a single multidimensional dataset that contains all of the metadata necessary to fully describe it.

By leveraging the NetCDF data format, MINC inherited an extensible format that allowed the creation of a rich and flexible set of metadata with arbitrary layout and size. Data and metadata are identified by textual names such as “image” or “history,” and each data object can have its own independent set of associated metadata (Rew and Davis, 1990). This allows the files to be self-documenting, and makes adding new fields straightforward.

Around the year 2001, it became clear that the situation with Analyze and related formats was becoming unwieldy. To address this, the NIH created the Neuroinformatics Technology Initiative (NIfTI) data format working group, which had as its first mandate the creation of a single, well-defined format for neuroimaging data. This format, known as NIfTI-1, is a set of extensions to the Analyze 7.5 format, and has been quite successful at resolving the major disputes over field usage and definitions. The specification also incorporates a mechanism for defining arbitrary extensions to the header.

Table 1 provides an informal comparison of some of the major features of different neuroinformatic data formats.

**Table 1:** **Brief summary of the characteristics of MINC and some other neuroinformatics data formats**.

**Format**	**Year**	**Files per 3D image**	**Richness of metadata**	**Human readability**	**Provenance support**
ACR-NEMA	1985	Typically many	High	Low	Low
Analyze 7.5	1986	Two	Low	Low	Low
DICOM	1992	Typically many	High	Low	Low
MINC	1992	One	High	High	High
NIfTI-1	2001	One	Moderate	Low	Moderate

At the same time that NIfTI-1 was being discussed, the development of MINC 2.0 began, with several important design goals. The major goal was to create a format with the strengths of the original MINC format, while allowing for future growth in the size and complexity of neuroimaging data.

## 3. Design and implementation

This section will detail the key design decisions in defining the structure of the MINC file and its associated metadata. Most of this information will be described with respect to the MINC 2.0 format, which is implemented using HDF5.

As the name implies, HDF5 is an inherently *hierarchical* format, in that each HDF5 file can contain a series of *groups* that can themselves contain other groups or *datasets* (The HDF Group, 1997–2015). HDF5 groups play a role similar to that of a directory in hierarchical file systems. MINC 2.0 takes full advantage of this hierarchy to maximize flexibility and to allow the coexistence of multiple data objects with explicit relationships. All HDF5 objects in a file are considered to be children of a “root” group, similar to the root directory in a POSIX filesystem.

### 3.1. Data representation and organization

Individual voxel data can be any of the most common machine data types. All voxels in an image are assumed to have the same type, typically signed or unsigned integers of 8, 16, or 32 bits. Voxel data may also be expressed in 32 or 64 bit floating-point format. Optionally, these may be restricted to a fixed subset of the natural range of the integer data type, to clearly specify unsigned 12-bit values, for example. NetCDF implemented this using a standard attribute, valid_range, which is a two-element vector defining the minimum and maximum valid values of the data. We used this mechanism in MINC 1.0 and have implemented the same in HDF5 as part of MINC 2.0.

While MINC 1.0 supported vector-valued voxels through use of an additional dimension, the MINC 2.0 extensions added support for complex numbers, multi-dimensional arrays, and enumerated (text label) types as voxel values. Of course, array types for voxels can be alternatively implemented as additional dimensions at the top level of the file.

As in all computational systems, multi-dimensional data is effectively stored as a contiguous vector of voxels, which are addressed by *voxel coordinates*. The data is logically stored in “row-major” order, meaning that in an *N*-dimensional file, voxels sharing the same addresses for dimensions 1..*N* − 1 are stored continuously. For example, the matrix:

$$\left[\begin{array}{lll} 10 & 12 & 14\\ 11 & 13 & 15 \end{array}\right]$$

would be stored in file or memory as:

$$\left[\begin{array}{llllll} 10 & 12 & 14 & 11 & 13 & 15 \end{array}\right]$$

HDF5 supports a number of file organization options that are also supported by MINC. Most significant is the capability to define a “chunked” data organization that splits the data into a series of limited-size blocks. The practical value of this is that it is straightforward to implement lossless compression on chunked data such that true random access can be maintained. This means that MINC tools and libraries can read individual parts of a file without loading and uncompressing the entire file, and that it is possible to read or write metadata without uncompressing any data. The HDF5 format also supports an error-checking algorithm that performs a checksum on each block. Recent versions of the MINC library can exploit this feature to guarantee the integrity of MINC data.

### 3.2. Hierarchical structure

**Figure 1:**
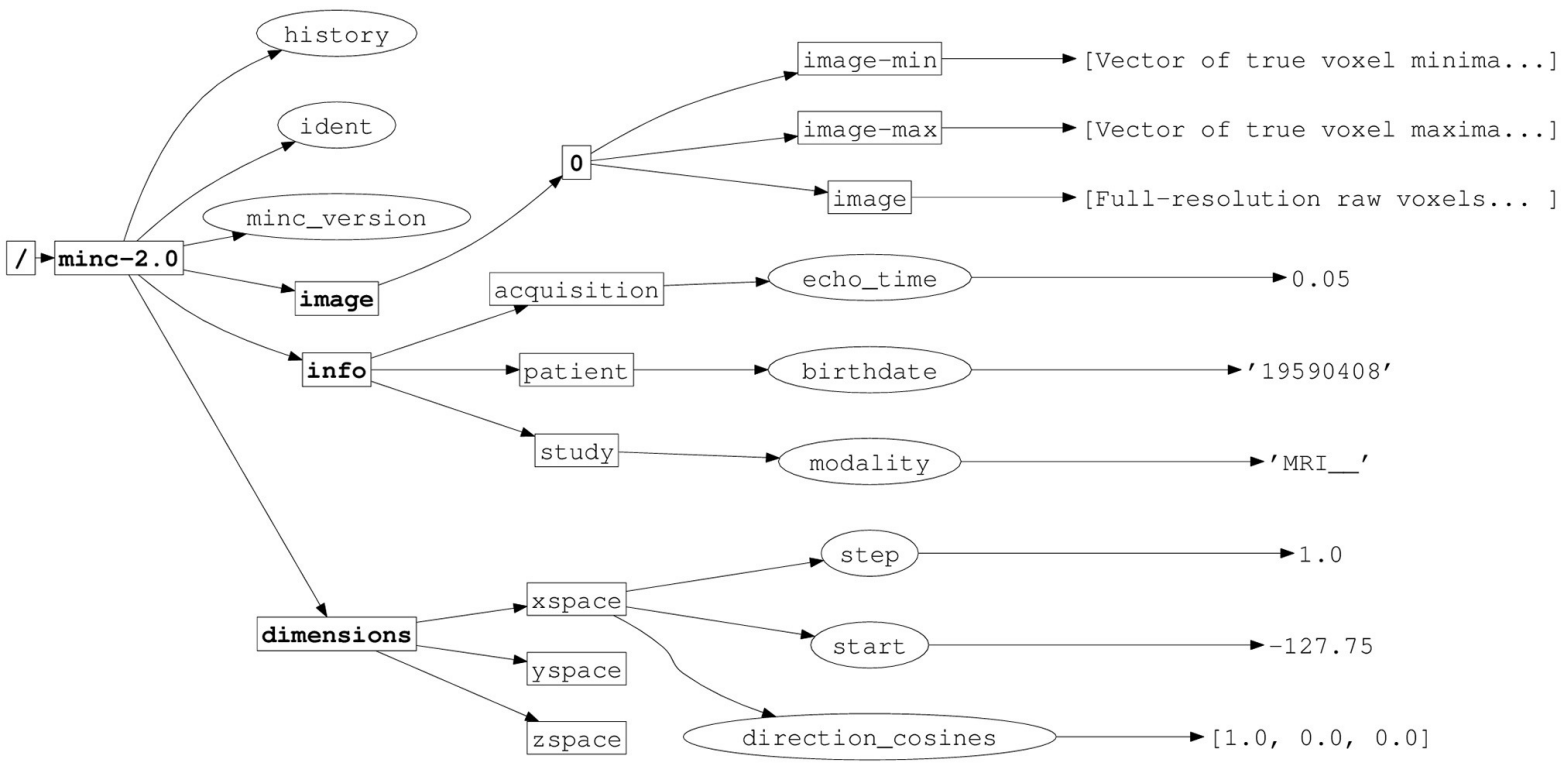
**Implementation of MINC 2.0 in HDF5, illustrating the hierarchical structure**. HDF5 groups have names in boldface, attributes are indicated with ellipses. Other rectangles indicate HDF5 datasets.

To allow MINC 2.0 data to coexist alongside other data structures in a single HDF5 file, and to differentiate MINC 2.0 files from other HDF5 files, a MINC group named minc-2.0 is created within the HDF5 root group. This group should be the only entry a MINC program creates in the HDF5 root group. All other MINC objects are created within (or “below”) the minc-2.0 group. This hierarchy is depicted in Figure 1.

There are exactly three subgroups within the minc-2.0 group: dimensions, image, and info.

Information about MINC 2.0 dimensions are placed in the subgroup called dimensions. This dimension information is further described in Section 3.4. It is considered global to all of the MINC objects in the file.

The image subgroup contains the actual image data of the MINC 2.0 file. Within this subgroup is at least one other subgroup named 0 (a single digit zero) corresponding to the maximum resolution data. The 0 group in turn contains three datasets, image, image-min, and image-max.

The MINC 2.0 informational variables (study, patient, etc.), are placed in the info subgroup. This subgroup is intended to be the repository of all ancillary data related to the scan, modality, experimental paradigm, and subject identification information. Typically, large datasets are not stored in this part of the hierarchy.

### 3.3. Metadata

Metadata in HDF5, and therefore in MINC, is supported through the use of named attributes that can contain arbitrary HDF5 data. Each HDF5 group or dataset can contain any number of metadata entries that further describe the interpretation of the data. The metadata can have any type, including integer, floating point, or character strings. Practical considerations limit these attributes to a size of a few thousand bytes, but there is currently no enforced limit to the size of an attribute (The HDF Group, 1997–2015).

Three standard attributes are located in the top-level of the MINC 2.0 hierarchy, within the minc-2.0 group. These are the attributes that are used to record global provenance information about the data:

- history - The processing history of this file. As a MINC file is created, most of the tools will use a library call to concatenate the current date and command line to the history of the first input file, and write this updated history to the output file.
- ident - A string that should uniquely identify this MINC file. In the standard library it is formed from a concatenation of the hostname, username, date and time, process id, and a global counter.
- minc_version - The version of the MINC library used to create this file.

There is a large group of descriptive metadata fields organized under the info group. The structures stored under this subgroup represent ancillary information which may be useful for provenance, statistical analysis, etc., but do not affect basic interpretation of data values or spatial coordinates. The details of these attributes are provided in Appendix 3.

For example, the patient dataset found within the info group is typically used to group information about the subject associated with the image. This would include the subject's name, age, weight, position, etc. The study dataset is used to group information about the overall experiment, including the modality, researcher name, scanner information, etc. Finally, the acquisition dataset groups details about the specific scanning parameters used, e.g., the echo time and repetition times for magnetic resonance images.

Each of these values may be queried or modified using one of several command-line tools. Specialized tools exist to interpret the MINC header, but generic HDF5 tools and libraries can be used as well.

### 3.4. Dimensions and coordinate system

The MINC libraries and file formats support an arbitrary number of dimensions. Dimensions are given names, which can be arbitrary strings. However, MINC files typically use some subset of five “standard” dimension names: xspace, yspace, zspace, time, and vector_dimension.

If a dimension is used in a particular file, its name will normally also define a dataset, known as a “dimension variable.” These dimension variables serve two purposes. Most importantly, they group the attributes associated with a given dimension. Secondarily, for an irregularly sampled dimension, they will contain a data vector that contains the values of the points at which this dimension was sampled. Note that irregularly-sampled dimensions also use *dimension width* variables that specify the width of each sample.

#### 3.4.1. Dimension ordering

While the number and ordering of dimensions is somewhat arbitrary, MINC files tend to follow certain conventions in practice. For example, if a file contains a time dimension, it should be the first, and therefore slowest-varying, dimension in the file, whereas a vector_dimension should be the final, fastest-varying dimension in the image. Spatial dimensions can be in arbitrary order, and in practice often are in one of either sagittal (XZY), coronal (YZX), or transverse (ZYX) organization. The final two spatial dimensions in the file dimensions in the file are termed the “image” dimensions, whereas the first spatial dimension is termed the “slice” dimension.

#### 3.4.2. Coordinate transformation

**Figure 2:**
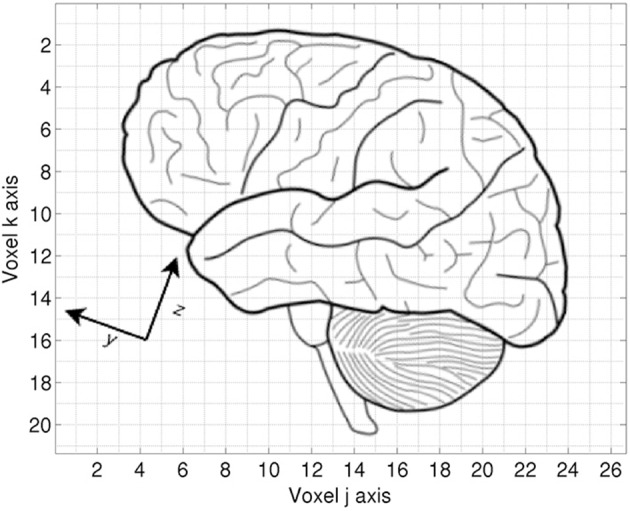
**Voxel vs. world coordinates**. Each grid square represents a single sample in the voxel space of the image. The voxel origin (0,0) would be in the upper left corner of the image. The world Y and Z directions are rotated 20⋅ relative to the voxel coordinates. The origin of the world coordinate system would be defined with respect to some anatomical landmark.

A distinction is made between the *voxel coordinates* and the *world coordinates*. The voxel coordinates are those assigned by the scanner's data collection, corresponding to the sampling grid of the volume. They are essentially integer values ranging from zero to one less than the number of data points along an axis. They are similar to the array indices used in most programming languages. In contrast, the world coordinates describe the actual orientation of the patient. MINC adopts the convention that the world X dimension increases from patient left to patient right, Y increases from patient posterior to anterior (back to front), and Z increases from patient inferior to superior. This relationship is illustrated in Figure 2.

#### 3.4.3. Dimension attributes

Each spatial dimension variable is associated with several attributes that define the relationship between the voxel and world coordinate systems. Historically, standard string values are padded with underscore characters to guarantee consistent length.

- direction_cosines - A 3-element floating point unit vector specifying the direction cosines of this axis. The default values are (1, 0, 0) for xspace, (0, 1, 0) for yspace, and (0, 0, 1) for zspace.
- start - A number specifying the world coordinate of the first position on this axis. It gives the position of the first voxel projected onto the axis, and thus is rotationally invariant. The default value is zero.
- step - A number specifying the length and sign of the unit vector along this axis, if the axis has regular spacing. If the axis has irregular spacing, this should be the mean step size. The step size can be negative to indicate reverse voxel orientation. The default value is 1.0.
- spacing - A string specifying whether the spacing along the axis is regular or irregular. The default value is regular__.
- length - An integer specifying the length in samples of this axis. This attribute is mandatory in MINC 2.0.
- alignment - A string specifying whether the coordinates are defined relative to the start, center, or end of a voxel. The default value is start_ for time dimensions or center for spatial dimensions.
- spacetype - A string specifying the type of coordinate system. Standard values are either native____, the spatial coordinates of the scanner, talairach_, the standardized coordinate system for the brain defined by Talairach and Tournoux (1988), or callosal__, a similar system used in epilepsy surgery (Lehman et al., 1992). The default is native____.
- filtertype - A string specifying the shape of the convolving filter. Currently, can be one of square____, gaussian__ or triangular. If this attribute is absent, a value of square____ should be assumed. Applies only to dimension width variables.
- units - A string that specifies the units of the dimension, typically “mm” (millimeters) for spatial dimensions and “s” (seconds) for time dimensions.
- width - A number giving the full-width half-maximum width of all samples for regularly sampled dimensions. It can be used for irregular widths to specify the average width. If this attribute is absent, a value of 1.0 should be assumed. Applies only to dimension width variables.

The direction cosines define the unit vector in the world coordinate space that corresponds to a step along the associated voxel dimension. The direction cosines in a MINC file always point along the positive axis. Reverse voxel orientation, if present, is specified by a negative step size.

See Appendix 1 for more details about the coordinate transformation.

By convention, the xspace, yspace, and zspace dimension variables are associated with the voxel dimension having the largest direction_cosines component along the world X, Y, or Z axis, respectively. However, this relationship is not an absolute requirement.

#### 3.4.4. Associating HDF5 dataspaces with MINC dimensions

NetCDF defines a named-dimension abstraction that permits a dimension and its length to be associated with a text symbol. This dimension may then be used to define any number of NetCDF variables. MINC 1.0 relies on this feature to link the dimensionality of related objects. Unfortunately, HDF5 does not implement a comparable dimension abstraction. Instead, all HDF5 data objects (datasets and attributes) are associated with an HDF5 construct called a “dataspace.” A dataspace in HDF5 may either be of one of three classes, “null,” “scalar,” or “simple.” Simple dataspaces consist of an ordered list of dimension lengths. Dataspaces in HDF5 are not global entities, and cannot be assigned symbolic names—every data object is associated with its own dataspace.

Since the structure of the dataspace alone is insufficient to allow software to discover the relationships between the dimensions of associated data objects, MINC 2.0 defines a dimorder attribute that makes these relationships explicit. Every data object that is non-scalar must have an associated dimorder attribute.

The dimorder attribute's value is a character string that consists of an ordered, comma-separated list of the mnemonic names associated with the dimension variables. This allows MINC 2.0 to emulate the named dimensions of NetCDF.

### 3.5. Voxel value scaling

Scanners often represent raw voxel data types as small integers in either 2's complement or unsigned integer format. However, there is often a natural mapping of these voxel values into a “true” intensity value, which is usually a simple linear scaling into some real interval. Furthermore, many scanners still acquire and save data in separate 2D slices such that each slice may have a different real range even if the individual voxels each have the same fixed precision. MINC 1.0 and 2.0 both permit the retention of the complete real range of the acquired data, even if each slice has a different mapping into the real range, without increasing the number of bits used for each voxel. This guarantees that the precision of the original data is fully represented in the MINC volume.

MINC accomplishes this scaling by creating two variables, image-min and image-max, which record the real interval into which raw voxel values should be mapped. These two variables may have dimensionality of up to the first *N* − 2 dimensions of the image. Thus, in a 4-dimensional functional image with transverse slices, the dimension order would be time, zspace, yspace, and xspace. The image-min and image-max variables could either be scalar, indicating a global scaling factor, or they could be vectors varying in time, or possibly matrices varying in time and zspace.

The scalar case defines a global transformation for all voxel values, similar to that used in the NIfTI-1 format.

In the most common case of per-slice scaling over a three-dimensional image, the mapping from the raw voxel value *v*_*r*_(*i, j, k*) to the true value *v*_*t*_(*i, j, k*) is calculated as:

(1) $$\begin{array}{l} v_{t}(i,j,k)=(v_{r}(i,j,k)-V_{min})\frac{\left(I_{max}(i)-I_{min}(i)\right)}{\left(V_{max}-V_{min}\right)}+I_{min}(i) \end{array}$$

where *I*_*max*_(*i*) and *I*_*min*_(*i*) are the values of the image-max and image-min variables and *V*_*max*_ and *V*_*min*_ are the values of the valid_range attribute.

Thus, in a typical file that defines a scalar image-max and image-min equal to 1.0 and 0.0, respectively, and a valid_range of [0, 4095], reflecting a 12-bit precision, the voxel value *v*_*r*_(*i, j, k*) = 410 would approximately map onto the true value *v*_*t*_(*i, j, k*) = 0.1. Values outside the valid_range represent invalid or unknown values.

### 3.6. Multi-resolution images

MINC 2.0 defines the possibility that a single file may contain multiple images that represent lower resolution versions of the original image. This enables precomputation of lower resolution data for visualization applications, for example. These images may be defined at any resolution of *s*2^*n*^ for any reasonable value of *n*, where *s* is the voxel resolution of the original image. Therefore, a MINC file might contain an original image at 0.1 mm resolution as well as additional images at 0.4 and 0.8 mm resolution.

This is implemented by adding additional groups to the hierarchy within the “image” group defined in Section 3.2. The names of these groups are derived from the appropriate value of *n*, that is, the log_2_ of the scaling factor applied to the voxel size in this subimage. Thinking of the structure as directories in a UNIX-like filesystem, the full-resolution image is stored at “/minc-2.0/image/0,” the half-resolution image, if present, is stored at “/minc-2.0/image/1,” quarter-resolution image would be at “/minc-2.0/image/2,” etc.

It is assumed that each of these subimages can use the same overall mapping from voxel to world coordinate systems, so that information is not replicated. However, each of these subimages will contain the image, image-min, and image-max datasets.

## 4. Example applications

MINC files are commonly used to represent any 2D or 3D image data, such as MRI, PET, CT, or histology. One advantage for PET data is MINC's ability to represent an irregularly-spaced time axis. MINC can also be used to represent derived data such as diffusion tensors and deformation fields.

In this section, we will give a few examples that highlight the applications that have been made easier by adopting the MINC format.

### 4.1. Diffusion MRI

The flexibility of the MINC format provided for easy extensions for use with diffusion MRI (dMRI). A typical diffusion image consists of multiple 3D images acquired using differing gradient fields (see for example Le Bihan, 2003). For post-processing it is necessary to record at least the intensity and direction of the gradient field for each acquired volume. These individual volumes are combined into a single MINC file by concatenating the volumes along the time dimension.

The flexibility of the MINC format facilitates the inclusion of the necessary metadata in the MINC header. Five attributes are added to the acquisition variable of the MINC header of a dMRI scan. These are:

1. acquisition:direction_x - A vector containing the X-components of each gradient field direction for each volume.
2. acquisition:direction_y - A vector containing the Y-components of each gradient field direction for each volume.
3. acquisition:direction_z - A vector containing the Z-components of each gradient field direction for each volume.
4. acquisition:bvalues - A vector containing the b-value associated with the gradient field for each volume.
5. acquisition:b_matrix - A vector containing the 6-component b-matrix associated with the gradient field for each volume.

The value of each of these attributes is a vector of floating-point values with the same length as the time dimension.

Maintaining this information in the MINC header, rather than requiring an auxiliary file, helps guarantee the consistency and traceability of research using dMRI data. These fields were defined based on informal discussions of researchers in the MINC community. Because these new fields do not interfere with or replace existing fields, these extensions required no other changes in the MINC version or header format.

### 4.2. Provenance

An increasing interest in the problem of image provenance has emerged in recent years (MacKenzie-Graham et al., 2008). Questions of provenance are paramount in complex, multi-center studies, but they can be important in smaller studies as well. Knowing exactly where, when, and how a file was processed, and what files it incorporates, is critical for addressing the reliability and reproducibility of results. It can also play a role in more mundane issues of technical support and debugging complex pipelines.

The creators of MINC anticipated these issues and accommodated them in both MINC 1.0 and MINC 2.0. From the early days of MINC 1.0, the header has included a global history attribute that records the processing history of a file. This includes the date and command line used for each processing step. While admittedly a limited view of provenance, even this relatively simple tool is invaluable in providing user support and diagnosing problems, because of the value of reconstructing the processing history of the file.

In MINC 2.0, the header also added both a version identifier that records the version of the MINC tools that created the file, and a unique identifier that encodes (amongst other things) the computer and user name responsible for creating the file, and the date and time of the file creation.

One example of the use of this flexibility is the inclusion of provenance information in the MINC header. Existing DICOM-to-MINC conversion tools preserve most of the DICOM fields in the MINC header. The convention is to create attributes within the info group named dicom_0xGGGG:el_0xEEEE where GGGG and EEEE are respectively the 4-digit hexidecimal DICOM group and element numbers, and the associated attribute values are generally stored unmodified.

The ability to store larger attributes means that it would be fairly easy to include larger pieces of information, such as processing logs, system configurations, or other provenance information. These could be stored in plain text, binary, or structured text such as JSON or XML.

### 4.3. Anonymization

Anonymization of data is important for protecting the privacy of study participants, while researchers need to retain enough non-identifying information for data analysis purposes.

The structure of the MINC header implies that most fields in the patient group can be removed from anonymized files. One approach would be to retain only the required fields for a given study, such as sex, age, or weight. In some cases it may also be desirable to modify or remove indirectly identifying information such as acquisition or study dates and times. And for multi-center studies it may be advisable to remove attributes that identify specific operators, institutions, or researchers.

The MINC command-line tools include support for examining and modifying metadata, which can be used to search for and remove identifying information, either manually or automatically.

Given MINC's flexiblility researchers, identifying information may be found in non-standard locations. Researchers may be tempted to add non-standard fields for study-specific purposes. Common MINC extensions such as the storage DICOM fields (described in the preceding section) may create additional instances of certain identifying fields. Care must be taken to remove identifying information in non-standard locations in the structure.

## 5. Discussion

The development of provenance systems for imaging data (and scientific data in general) continues to attract interest (MacKenzie-Graham et al., 2008). Arguably, it is clearer to have as much provenance information located within the file as possible. Because of the limitations of existing data formats, it can be difficult to incorporate provenance data into these formats, whereas it is straightforward in MINC.

There are some disadvantages inherent in complex formats like HDF5. It is relatively challenging to read an HDF5 file “on the fly” over a streaming interface, as one cannot generally count on data being stored in a specific order. It is also more difficult to port relatively complex formats to new platforms. We have begun to address this by implementing a new library that can load MINC 1.0 and 2.0 format files within a web browser, for web-based applications such as visualization with BrainBrowser (Sherif et al., 2015).

Software supporting MINC has reached a high level of maturity and achieved broad adoption within parts of the neuroimaging community. A number of libraries provide support for a range of programming and image processing environments, such as Python, R, C/C++, and ITK. Several other platforms, including recent versions of Matlab, contain built-in HDF5 support. The MINC libraries provide both low-level and high-level access to the data and metadata of MINC files, with performance similar to that of the underlying HDF5 format. The basic command line tools include support for resampling/reshaping files, querying metadata, general voxel math, statistical operations, and comparison statistics, amongst others. More complex tools are available for functions such as automatic image registration, tissue classification, and visualization.

Simple extensions have allowed the MINC 2.0 header to grow over time to include information necessary for new modalities, without requiring a new or incompatible format. MINC's flexibility makes it possible to store an unlimited range of data types in a MINC file. Incorporating new data types such as genetic, geometric, or electrophysiological measurements is straightforward. For example, projects under consideration include the definition of formats for surface and connectivity information that could co-exist with the voxel image data in a MINC file. In addition, the flexible metadata format allows the inclusion of arbitrary supplementary information. The use of text names for metadata attributes renders them much more readily interpretable, while retaining the relative storage efficiency of a binary format.

One useful tool which MINC does not yet incorporate is a comprehensive validator for the format. Such a tool would check a file for the presence of all mandatory fields, and verify the consistency of the information in those fields. The authors hope to develop such a tool in the near future.

The flexibility of MINC and HDF5 will allow for interoperability between MINC and other neuroimaging standardization initiatives. The MINC community has defined potential additions to the format to support the Brain Imaging Data Structure (BIDS) initiative (Gorgolewski et al., 2016). We are also participating in the Human Atlas Working Group (Poline et al., 2016) and intend to incorporate compatible extensions for atlas support in MINC.

## Author contributions

RV wrote the initial draft of the manuscript, with much assistance from NK. PN designed and created the original MINC 1.0 format, with assistance from DC, AZ, and DM. RV, JS, and LB designed and implemented the MINC 2.0 format. VF, AJ, JL, and SR provided major support for enhancing and maintaining MINC. All authors reviewed and contributed to the final manuscript.

### Conflict of interest statement

The authors declare that the research was conducted in the absence of any commercial or financial relationships that could be construed as a potential conflict of interest.
