# Supplementary material for: MINC 2.0: A Flexible Format for Multi-Modal Images
Source: Front Neuroinform. 2016 Aug 11;10:35. doi: 10.3389/fninf.2016.00035 (PMC4980430; doi:10.3389/fninf.2016.00035)
Supplement: Supplementary file 1 [file DataSheet1.pdf]

1 DETAILS OF THE COORDINATE TRANSFORMATION

The direction cosines for spatial axis i is a normalized vector in three space such that:

$$\vec{c_i} = (c_{ix}, c_{iy}, c_{iz}). \tag{1}$$

Using δ_i to represent the value of the `step` attribute for spatial axis i, and defining s_i similarly for the `start` attribute, the vector $\vec{v_i}$ between adjacent elements along that axis is:

$$\vec{v_i} = \delta_i \, \vec{c_i} \tag{2}$$

and the origin $(0, 0, 0)$ in voxel coordinates is located at world coordinates (o_x, o_y, o_z) as given by:

$$\begin{bmatrix} o_x \\ o_y \\ o_z \end{bmatrix} = \begin{bmatrix} c_{ix} & c_{jx} & c_{kx} \\ c_{iy} & c_{jy} & c_{ky} \\ c_{iz} & c_{jz} & c_{kz} \end{bmatrix} \begin{bmatrix} s_i \\ s_j \\ s_k \end{bmatrix} \tag{3}$$

Finally, any homogeneous coordinate in voxel space $(v_x, v_y, v_z, 1)$ can be transformed to its equivalent in world space using the equation:

$$\begin{bmatrix} w_x \\ w_y \\ w_z \\ w_w \end{bmatrix} = \begin{bmatrix} c_{ix}\delta_i & c_{jx}\delta_j & c_{kx}\delta_k & o_x \\ c_{iy}\delta_i & c_{jy}\delta_j & c_{ky}\delta_k & o_y \\ c_{iz}\delta_i & c_{jz}\delta_j & c_{kz}\delta_k & o_z \\ 0 & 0 & 0 & 1 \end{bmatrix} \begin{bmatrix} v_i \\ v_j \\ v_k \\ 1 \end{bmatrix} \tag{4}$$

2 MINC 2.0 STRUCTURAL ATTRIBUTES

These attributes serve an supporting role in the definition of the MINC file itself. Many of them were required for MINC 1.0, but are no longer truly needed in MINC 2.0. They have been retained for MINC 2.0 for reasons of backward compatibility.

Each of these attributes are mandatory for any MINC standard variable, with the following exceptions:

- The `signtype` attribute is required only for variables of integral type.
- The `parent children` attributes are required only for variables of class `group` or `var-attribute`.

These seven attributes are considered structural:

- `children` - List of the names of the variables that are considered children of this variable. The list is separated by newline characters.
- `comments` - A string providing supporting information about the MINC variable's meaning or interpretation.
- `dimorder` - A string identifying the names and order of the dimension variables associated with the dimensions of an HDF5 dataset. The elements are separated with a comma. For example, the `image` dataset for a transverse 3D image might have a `dimorder` attribute with the value `"zspace,yspace,xspace"`. This attribute was introduced in MINC 2.0.

- `parent` - A string identifying by name the parent variable of this variable. If the string is empty (of length zero), the variable is at the root of the hierarchy.
- `signtype` - Because MINC 1.0, unlike NetCDF, differentiates between signed and unsigned integral types, this attribute is required to define the format of variables that store data of an integral type. The string value must be either `signed__` or `unsigned`. For 8-bit integers, the default is unsigned, for all other integer types the default is signed.
- `varid` - A string that identifies the variable's relationship to the MINC specification. All MINC standard variables should set this attribute to "MINC standard variable". Non-standard variables may either ignore this attribute or set it to some other string value.
- `vartype` - A string identifying the class of the variable. It must be one of `group________`, `dimension____`, `dim-width____`, or `var-attribute`.
- `version` - A string identifying the version of this variable. This string is always set to "MINC Version 1.0" for MINC standard variables.

In practice, only the `signtype` and `dimorder` attributes are important to the correct interpretation of the MINC 2.0 file structure, the remaining fields are retained for informational purposes only.

3 MINC 2.0 INFORMATIONAL ATTRIBUTES

These attributes serve an informational role in that they provide supporting information about the image, study, or patient. The following lists define the most widely-used fields, some conversion tools may incorporate additional information. Unless otherwise specified, numbers are stored in double-precision floating-point format.

3.1 `image` variable attributes

These attributes provide additional information about the state and type of the image data contained in the MINC 2.0 file.

- `complete` - A boolean attribute (with values `true_` or `false`) that indicates whether the variable has been written in its entirety. This can be used to detect program failure when writing out images as they are processed : complete is set to false at the beginning and set to true_ when all data has been processed and written to the file.
- `valid_range` - A vector of two numbers that specifies both the minimum and maximum valid values for this variable. Values outside this range must be treated as missing or uninitialized. In MINC, the order of the two values in the vector is not significant. This attribute should be considered mandatory for the MINC image variable, unless the variable uses the default range, which is defined to be the full range of an (possibly signed or unsigned) integer type or the interval (0.0, 1.0) for floating point types. It is not required for any other MINC variable.

3.2 `study` variable attributes

These attributes provide ancillary information about the study for which the image was collected. The attributes are grouped with the study variable for purposes of namespace organization, and are modeled after ACR-NEMA conventions (**ACR-NEMA committee**, 1988). Specific DICOM fields are cited by group and element number where appropriate. All of these attributes are optional, and have no default value defined.

- `admitting_diagnosis` - A string description of the admitting diagnosis.
- `attending_physician` - A string giving the name of the physician administering the examination.
- `department` - A string identifying the department conducting the study.
- `device_model` - A string specifying the model of the imaging device.
- `institution` - A string identifying the institution conducting the study.
- `manufacturer` - A string specifying the name of the imaging device manufacturer.
- `modality` - A string that represents the imaging modality used, typically one of: `PET__`, `SPECT`, `GAMMA`, `MRI__`, `MRS__`, `MRA__`, `CT___`, `DSA__`, `DR___`, or `label`.
- `operator` - A string giving the name of the operator of the imaging device.
- `procedure` - A string description of the procedure employed.
- `radiologist` - A string giving the name of the radiologist interpreting the examination.
- `referring_physician` - A string giving the name of the patient's primary physician.
- `start_time` - A string giving the time and date of the start of the study, in the format YYYYMMDD HHMMSS.FFFFFF, where FFFFFF is an string representation of fractional seconds. Either the entire time representation, or the fractional seconds, may be omitted if not required.
- `station_id` - A string identifying the specific imaging system that generated the image.
- `study_id` - A string identifying the study.

3.3 `patient` variable attributes

These attributes, which provide identifying information about the patient or subject, are grouped with the patient variable for purposes of namespace organization. They are modeled after ACR-NEMA (**ACR-NEMA committee**, 1988) conventions for patient identification. All of these attributes are optional, and have no default value defined.

- `address` - A string giving the patient's address.
- `age` - A number giving the patient's age in years.
- `birthdate` - A string specifying the patient's birthdate in the form YYYYMMDD.
- `full_name` - A string specifying the full name of the patient.
- `identification` - A string specifying identification information for the patient.
- `insurance_id` - A string giving the patient's insurance plan id.
- `other_ids` - A string giving other identification information for the patient.
- `other_names` - A string giving other names for the patient.
- `position` - A string giving the patient's position. See DICOM `(0018,5100)`.
- `sex` - A string specifying the patient's sex: `male__`, `female` or `other_`.
- `size` - A number giving the patient's height or length in meters.
- `weight` - A number giving the patient's weight in kilograms.

3.4 `acquisition` variable attributes

These attributes store parameters about the acquisition. All of these attributes are optional, and have no default value defined.

- acquisition_id - A string identifying this acquisition in the overall set of acquisitions.
- acquisition_time - A string giving the time of this particular acquisition, in the same format as study:start_time.
- contrast_agent String identifying the contrast or bolus agent.
- dose_units - A string giving units of dose.
- echo_number - A number giving the echo number of this acquisition
- echo_time - A number giving the time in seconds between the middle of a 90 degree pulse and the middle of spin echo production.
- echo_train_length - A number giving the number of echoes. See DICOM (0018,0091).
- flip_angle - Floating-point number specifying the number of degrees of rotation between the net magnetization vector and the main magnetic field.
- image_time - A string giving the time of this particular image.
- image_type - A string giving the type of this image. See DICOM (0008,0008).
- imaged_nucleus - A string specifying the nucleus that is resonant at the imaging frequency.
- imaging_frequency - A number giving the precession frequency in Hz of the imaged nucleus.
- injection_length - A number giving the time duration of the injection (in seconds).
- injection_time - A string giving time (and date) of injection, in the same format as study:start_time.
- injection_dose - Total dose of radionuclide or contrast agent injected (in units specified by dose_units).
- injection_route - A string identifying administration route of injection.
- injection_volume - A number giving the volume of injection in milliliters.
- inversion_time - A number giving the time in seconds after the middle of the inverting RF pulse to the middle of the 90 degree pulse to detect the amount of longitudinal magnetization.
- mr_acq_type - A string identifying the spatial data encoding method, e.g. "2D" or "3D". See DICOM (0018,0023).
- num_averages - A number of times a given pulse sequence is repeated before any parameter is changed.
- num_phase_enc_steps - A number of phase encoding steps in this MR acquisition. See DICOM (0018,0089).
- num_slices - A number of images in this acquisition. See DICOM (0020,1002).
- percent_phase_fov - A number giving the percentage of the field of view in the phase direction compared with the field of view in the frequency direction. See DICOM (0018,0092).
- percent_sampling - A number giving the percentage of acquisition matrix lines sampled. See DICOM (0018,0093).
- phase_enc_dir - A string specifying the direction of phase encoding, either "ROW" or "COL". See DICOM (0018,1312).
- pixel_bandwidth - A number specifying the inverse of the sampling period, in hertz per pixel. See DICOM (0018,0095).
- protocol - A string description of the protocol for image acquisition.

- `radionuclide` - A string specifying the isotope administered.
- `radionuclide_halflife` - A number giving the half-life of the radionuclide in seconds.
- `receive_coil` - A string giving the name of the receive coil used in an MR acquisition.
- `repetition_time` - A number giving the time in seconds between pulse sequences.
- `scanning_sequence` - A string description of type of data taken.
- `series_description` - A string giving the text description of the series.
- `series_time` - A string giving the start time of this series, in the same format as `study:start_time`.
- `slice_order` - A string specifying the order of acquisition of individual slices, one of `descending`, `ascending`, or `interleaved`.
- `SAR` - A number specifying the specific absorption rate in watts per kilogram.
- `slice_thickness` - A number giving the slice thickness in millimeters.
- `start_time` - A string giving the start date and time for the overall acquisition, in the same format as `study:start_time`.
- `tracer` - A string identifying tracer labeled with radionuclide that was injected.
- `transmit_coil` - A string identifying the transmit coil used in an MR acquisition.
- `window_center` - A number specifying a linear conversion for the pixels. See DICOM `(0020,1050)`.
- `window_width` - A number specifying a linear conversion for the pixels. See DICOM `(0020,1051)`.

3.5 Dimension variable attributes

All attributes used by both dimension and dimension-width variables are described in Section 3.4.3.

4 DETAILED HEADER EXAMPLE

A standard HDF5 tool can be used to list each of the groups and datasets in a MINC 2.0 HDF5 file:

```
/                         Group
/minc-2.0                 Group
/minc-2.0/dimensions      Group
/minc-2.0/dimensions/xspace Dataset {176}
/minc-2.0/dimensions/yspace Dataset {SCALAR}
/minc-2.0/dimensions/zspace Dataset {SCALAR}
/minc-2.0/image           Group
/minc-2.0/image/0         Group
/minc-2.0/image/0/image   Dataset {176, 256, 256}
/minc-2.0/image/0/image-max Dataset {176}
/minc-2.0/image/0/image-min Dataset {176}
/minc-2.0/info            Group
/minc-2.0/info/acquisition Dataset {SCALAR}
/minc-2.0/info/dicom_0x0008 Dataset {SCALAR}
...
```

```
/minc-2.0/info/dicom_0x0040 Dataset {SCALAR}
/minc-2.0/info/dicom_0x7fe0 Dataset {SCALAR}
...
/minc-2.0/info/patient   Dataset {SCALAR}
/minc-2.0/info/study     Dataset {SCALAR}
```

Specifically note that in this image, the `xspace` dimension variable is a vector of length 176, containing specific sampling points for each of the slices. The only image present is at full-resolution under the path `/minc-2.0/image/0`. Both the `image-min` and `image-max` variables are present, and specify per-slice scaling values over the raw voxel data in the image. Note also that most of the DICOM fields from the original conversion are preserved in the various `dicom_0xGGGG` groups. This allows MINC to carry the maximum possible amount of provenance data.

The `history` value has the form:

```
ATTRIBUTE "/minc-2.0/history" {
   ...
   DATA {
   (0): "Wed Dec  8 17:49:07 2004>>> dicomserver
         Fri Aug 14 12:39:08 2015>>> /home/rvincent/Documents/BIC/
minc-toolkit/build-debug/minctools/progs/mincconvert -2 /data1/users/
bert/nfs-bert/vincent_robert_20041208_165128_2_mri.mnc.gz vincentr.mnc"
```

This specifies that the file was created (on the Montreal Neurological Institute's DICOM server) on December 8th, 2004, and was converted to MINC 2.0 format on August, 14th 2015.

Examining the DICOM attributes, we see that the header also includes the complete 13060-character ASCII representation of the MRI scanning parameters produced by many Siemens scanners:

```
ATTRIBUTE "/minc-2.0/info/dicom_0x0023/el_0x0006" {
   DATATYPE  H5T_STD_U8LE
   DATASPACE  SIMPLE { ( 13060 ) / ( 13060 ) }
   DATA {
   (0): 35, 35, 35, 32, 65, 83, 67, 67, 79, 78, 86, 32, 66, ...
   (17): 32, 35, 35, 35, 10, 117, 108, 86, 101, 114, 115,   ...
   ...
   (13031): 86, 97, 108, 105, 100, 32, 32, 32, 32, 32, 32,  ...
   (13046): 32, 32, 32, 32, 32, 32, 32, 32, 32, 61, 32, 49, 10, 32
   }
}
```

In summary, the flexibility of the header permits storage of large, arbitrarily formatted information. This can be very relevant to proper provenance implementations.

5 FOR MORE INFORMATION

More information about the MINC format and software is available at the following URLs:

- https://en.wikibooks.org/wiki/MINC - Documentation on the MINC format, library specification, and basic tools.
- http://bic-mni.github.io/ - Binary releases of the MINC tools and documentation about additional MINC-aware tools.
- https://github.com/BIC-MNI/libminc - Source code of the core MINC libraries.
- https://github.com/BIC-MNI/minctools - Source code of the basic MINC command-line tools.
- http://www.bic.mni.mcgill.ca/mailman/listinfo/minc-users - Mailing list for discussion of MINC questions, ideas, and extensions.

REFERENCES

ACR-NEMA committee (1988), Digital imaging and communication, ftp://medical.nema.org/medical/dicom/1988/ACR-NEMA_300-1988.pdf
